# Fluid management in septic patients with pulmonary hypertension, review of the literature

**DOI:** 10.3389/fcvm.2023.1096871

**Published:** 2023-03-02

**Authors:** Blerina Asllanaj, Elizabeth Benge, Jieun Bae, Yi McWhorter

**Affiliations:** ^1^Department of Internal Medicine, HCA Healthcare, MountainView Hospital, Las Vegas, NV, United States; ^2^Kirk Kerkorian School of Medicine at UNLV, Las Vegas, NV, United States; ^3^Department of Critical Care Medicine, HCA Healthcare, MountainView Hospital, Las Vegas, NV, United States

**Keywords:** pulmonary hypertension, sepsis, intravenous fluid, hemodynamics, pulmonary circulation

## Abstract

The management of sepsis in patients with pulmonary hypertension (PH) is challenging due to significant conflicting goals of management and complex hemodynamics. As PH progresses, the ability of right heart to perfuse lungs at a normal central venous pressure (CVP) is impaired. Elevated pulmonary vascular pressure, due to pulmonary vasoconstriction and vascular remodeling, opposes blood flow through lungs thus limiting the ability of right ventricle (RV) to increase cardiac output (CO) and maintain adequate oxygen delivery to tissue. In sepsis without PH, avoidance of volume depletion with intravascular volume replacement, followed by vasopressor therapy if hypoperfusion persists, remains the cornerstone of therapy. Intravenous fluid (IVF) resuscitation based on individualized hemodynamic assessment can help improve the prognosis of critically ill patients. This is accomplished by optimizing CO by maintaining adequate preload, afterload and contractility. Particular challenges in patients with PH include RV failure as a result of pressure and volume overload, gas exchange abnormalities, and managing IVF and diuretic use. Suggested approaches to remedy these difficulties include early recognition of symptoms associated with pressure and volume overload, intravascular volume management strategies and serial lab monitoring to assess electrolytes and renal function.

## Introduction

Pulmonary hypertension (PH) comprises five World Health Organization (WHO) classes with numerous disease states leading to increasing pressure within the pulmonary circulation and ultimately leading to right heart failure. Patients with PH and right ventricle (RV) failure are frequently encountered in clinical practice as well as critical illness. The treatment of sepsis in patients with PH is challenging. The metabolic demand of sepsis often requires supra-physiologic cardiac output (CO) to maintain end-organ perfusion. This would unmask RV failure in patients with underlying PH. Management of RV failure involves optimization of preload and maintenance of systemic blood pressure (1). Resuscitation with end goals to restore intravascular volume and oxygen delivery has become gold standard since adoption of early goal-directed therapy (EGDT) and the development of the Surviving Sepsis Campaign Guidelines (2–4). Critically-ill patients with PH require a diligent approach to fluid management due to potential hemodynamic decompensation. A hemodynamically guided conservative application to fluid therapy in patients with sepsis and PH would be prudent and likely to improve the outcome of this disease. We reviewed current practices to determine the influence of fluid management on the outcomes of septic patients with PH.

## Overview of normal cardiovascular physiology

**Figure 1:**
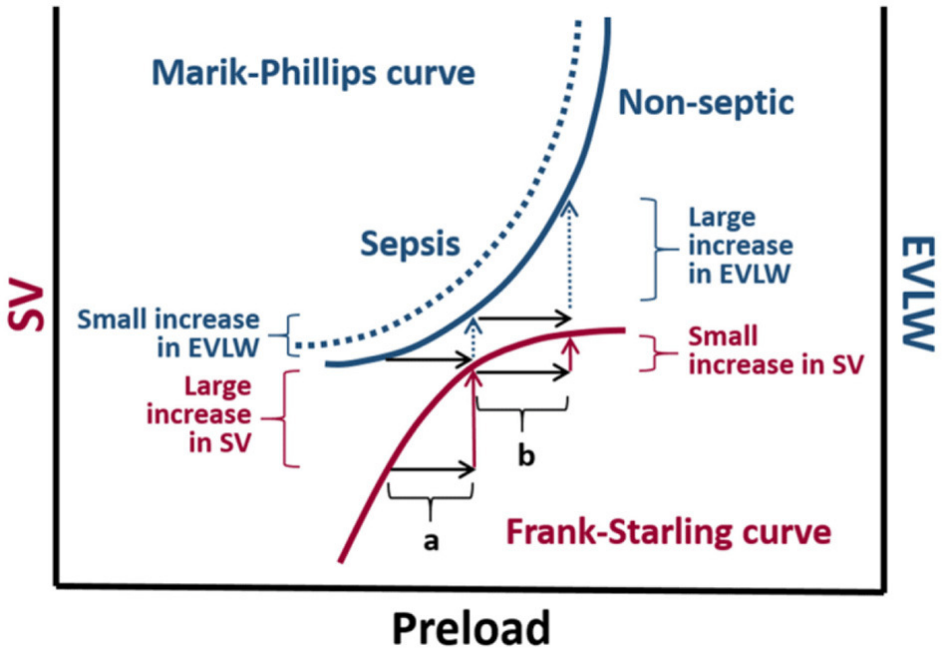
The Marik-Phillips and Frank-Starling curves correlating changes in extra vascular lung water (EVLW) and stroke volume (SV) with preload. For cardiac function correlating to the ascending limb of the Frank-Starling curve, an increase in preload induced by a fluid challenge (a) does not substantially increase EVLW. If a fluid challenge is administered to individuals whose heart is operating on the flat portion of the Frank-Starling curve, the increase in preload (b) may result in a large increase in EVLW. Due to endothelial glycocalyx damage associated with sepsis, larger increases in EVLW can be expected in septic individuals (dashed curve) (9, 10).

The concept that CO is strictly dependent on stroke volume (SV) was popularized by Frank-Starling (5). Venous return refers to the blood flow from peripheral circulation to the right atrium, and except for periods of a few seconds, it is equal to CO. Guyton recognized the importance of determining the role of both mean systemic pressure and right atrial pressure (RAP) in controlling venous return, and measuring both accurately proved to be difficult (6). RAP, also known as CVP is normally 1–6 mmHg. Mean systemic pressure, normally in the range of 7–10 mmHg, is affected by blood volume and vascular tone. Due to the restraining effects of the pericardium, diastolic compliance of the heart reduces as volume increases. The gradient for venous return is decreased as the CVP is increased with large volume fluid resuscitation (7). Therapeutic interventions and compensatory mechanisms can regulate venous return and CO to appropriate values. The Frank-Starling relationship is an intrinsic property of the heart by which increased left ventricular end-diastolic volume results in enhanced left ventricular SV (8) (Figure 1). Fluid administration can optimize preload if the increase in mean systemic pressure is greater than CVP and both ventricles are functional. However, if either condition is not satisfied, pulmonary venous congestion exceeding critical left ventricular end diastolic pressure (LVEDP) may result pathologically.

## Cardiopulmonary hemodynamics in pulmonary hypertension

### Definition and classification of pulmonary hypertension

PH demonstrates a pathological sign of hemodynamic remodeling of various clinical conditions. The 5th World Symposium on PH classified it into five groups of disorders (Table 1). PH is defined by a mean pulmonary artery pressure (PAP) >20 mmHg at rest for all the clinical groups, confirmed measurement during right heart catheterization (11). It is also classified as pre-capillary, isolated post-capillary, and mixed pre- and post-capillary based on hemodynamic data (12). Pre-capillary PH is attributed to primary elevation of pressure in the pulmonary arterial vasculature, such as seen in clinical group 1. Post-capillary PH is the result of elevations in the pulmonary venous and capillary systems, with pulmonary capillary wedge pressure (PCWP) > 15 mmHg, attributed to left heart disease (group 2) or multifactorial mechanisms (group 5) (13). The 6th World Symposium on PH, recognizing the possibility of mixed pre- and post-capillary PH, incorporated pulmonary vascular resistance (PVR) >3 woods unit (WU) into the definition of PH (Table 2) with slight difference in ESC guideline of PVR >2 WU (Table 3) (11, 14). The inclusion of PVR emphasized the importance of making the distinction between pre- and post-capillary based on the PCWP and PVR (14, 15). At the present time, 1% of the global population is diagnosed with PH, with WHO group 2 being most common (11).

**Table 1:** 2022 ESC clinical classification of pulmonary hypertension (11).

**Group 1 pulmonary arterial hypertension (PAH)**
1.1 Idiopathic
1.1.1 Non-responders at vasoreactivity testing
1.1.2 Acute responders at vasoreactivity testing
1.2 Heritable^a^
1.3 Associated with drugs and toxins^a^
1.4 Associated with:
1.4.1 Connective tissue disease
1.4.2 HIV infection
1.4.3 Portal hypertension
1.4.4 Congenital heart disease
1.4.5 Schistosomiasis
1.5 PAH with features of venous/capillary (PVOD/PCH) involvement
1.6 Persistent PH of the newborn
**Group 2 PH associated with left heart disease**
2.1 Heart failure
2.1.1 with preserved ejection fraction
2.1.2 with reduced or mildly reduced ejection fraction^b^
2.2 Valvular heart disease
2.3 Congenital/acquired cardiovascular conditions leading to post-capillary PH
**Group 3 PH associated with lung diseases and/or hypoxia**
3.1 Obstructive lung disease or emphysema
3.2 Restrictive lung disease
3.3 Lung disease with mixed restrictive/obstructive pattern
3.4 Hypoventilation syndromes
3.5 Hypoxia without lung disease (e.g., high altitude)
3.6 Developmental lung disorders
**Group 4 PH associated with pulmonary artery obstructions**
4.1 Chronic thrombo-embolic PH
4.2 Other pulmonary artery obstructions^c^
**Group 5 PH with unclear and/or multifactorial mechanisms**
5.1 Hematological disorders^d^
5.2 Systemic disorders^e^
5.3 Metabolic disorders^f^
5.4 Chronic renal failure with or without hemodialysis
5.5 Pulmonary tumor thrombotic microangiopathy
5.6 Fibrosing mediastinitis

HIV, human immunodeficiency; PAH, pulmonary arterial hypertension; PCH, pulmonary capillary hemangiomatosis; PH, pulmonary hypertension; PVOD, pulmonary veno-occlusive disease.

a Patients with heritable PAH or PAH associated with drugs and toxins might be acute responders.

b Left ventricular ejection fraction for HF with reduced ejection fraction: <40%; for HF with mildly reduced ejection fraction: 41–49%.

c Other causes of pulmonary artery obstructions include: sarcomas (high or intermediate grade or angiosarcoma), other malignant tumors (e.g., renal carcinoma, uterine carcinoma, germ-cell tumors of the testis), non-malignant tumors (e.g., uterine leiomyoma), arteritis without connective tissue disease, congenital pulmonary arterial stenosis, and hydatidosis.

d Including inherited and acquired chronic hemolytic anemia and chronic myeloproliferative disorders.

e Including sarcoidosis, pulmonary Langerhans' cell histiocytosis, and neurofibromatosis type 1.

f Including glycogen storage diseases and Gaucher disease.

**Table 2:** The 6th world symposium defined three hemodynamic profiles of pulmonary hypertension (14).

**Classification**	**Mean pulmonary artery pressure**	**Pulmonary capillary wedge pressure**	**Pulmonary vascular resistance**
Isolated pre-capillary PH	>20 mmHg	< 15 mmHg	>3 WU
Combined pre- and post-capillary PH		>15 mmHg	>3 WU
Isolated post-capillary PH		>15 mmHg	< 3 WU

WU, Wood units.

**Table 3:** ESC guidelines on PH definition based on hemodynamic characteristics (11).

**Hemodynamic definitions of pulmonary hypertension**	
**Definition**	**Hemodynamic characteristics**
PH	mPAP >20 mmHg
Pre-capillary PH	• mPAP >20 mmHg • PAWP <15 mmHg • PVR >2 WU
IpcPH	• mPAP >20 mmHg • PAWP >15 mmHg • PVR <2 WU
CpcPH	• mPAP >20 mmHg • PAWP >15 mmHg • PVR >2 WU
Exercise PH	mPAP/CO slope between rest and exercise >3 mmHg/L/min

CO, cardiac output; CpcPH, combined post- and pre-capillary pulmonary hypertension; IpcPH, isolated post-capillary pulmonary hypertension; mPAP, mean pulmonary arterial pressure; PAWP, pulmonary arterial wedge pressure; PH, pulmonary hypertension; PVR, pulmonary vascular resistance; WU, Wood units. Some patients present with elevated mPAP (>20 mmHg) but low PVR (<2 WU) and low PAWP (<15 mmHg); this hemodynamic condition may be described by the term ‘unclassified PH.

### Pathophysiology and structural heart disease associated with pulmonary hypertension

Pathological features which characterize the diverse PH groups are evaluated using a constellation of non-invasive imaging and clinical findings. In pre-capillary PH, progression of disease, along with increased PVR and PAP, will result in overload of RV pressure leading to RV hypertrophy (homeometric adaptation) (16). To maintain CO, heterometric adaptation also occurs, resulting in right heart systolic dysfunction through progressive dilation of RV (17). In addition, right heart diastolic dysfunction occurs as a result of increasing fibrosis and impaired contractility of the RV sarcomeres (18). Subsequently, the chronic changes observed in RV will affect the structure and function of left ventricle (LV). As described by Bernheim, this ventricular interdependence is attributed to the constraints of the pericardium (19). The enlarged RV chamber size and wall thickness cause bowing of the interventricular septum and compression of LV. The decrease in LV chamber size due to the constraints of the pericardium result in decreased filling, compliance and SV (20). Consequently, the diminished CO elucidates the signs and symptoms of PH such as hypotension, peripheral edema and dyspnea.

### Diagnostic evaluation of pulmonary hypertension

Although right heart catheterization is considered the gold standard for diagnosis of PH, estimation of pulmonary pressure *via* echocardiography plays a seminal role in detection of PH in initial stages owing to its wide application and non-invasive nature. Transthoracic echocardiography (TTE) evaluates both RV and LV structure and function. Cardiac evaluation by TTE also includes the probability of PH using the tricuspid regurgitant jet velocity (TRV) to estimate the pulmonary artery systolic pressure (ePASP). The most recent guidelines issued by the European Society of Cardiology (ESC) in 2022 endorse using the peak TRV, rather than ePASP, to report the probability of PH. A peak TRV above 2.8 m/s suggests the probability of PH echocardiographically based on updated hemodynamic definition (11). Many clinicians still use the 2009 and 2015 guidelines issued by ESC that suggest the possibility of PH if ePASP is >36 mmHg and the TRV is >2.9 m/s with findings suggestive of PH on TTE (21, 22). Sustained elevation of ePASP will result in hypertrophy and dilatation of RV resulting in cor pulmonale for patients with group 3 PH (23).

ECG (electrocardiogram) findings have been recently incorporated in predictive score models to differentiate between pre- and post-capillary PH (24). Atrial fibrillation and signs of left ventricular hypertrophy are more likely on ECG in post-capillary PH whereas right axis, sinus rhythm, ST-T segment depression and T-wave inversion in the right precordial and inferior leads are most frequently seen in pre-capillary PH (25). In PH, enlargement of right atrium (RA) is indicative of advancing disease and potential progression to right heart failure as elevated pulmonary and right ventricular pressures are transmitted to RA (26). Chronic RA pressure overload and stretching, coupled with chronic hypoxia, alter the atrial substrate by promoting fibrosis, which predispose to a risk for atrial fibrillation and atrial flutter (27). This is consistent with emerging data demonstrating atrial fibrosis in the pathogenesis of atrial fibrillation as demonstrated by delayed enhancement on MRI (28).

N-terminal pro-brain natriuretic peptide (NT-proBNP) may be elevated in atrial fibrillation as it's secreted from the cardiomyocytes of both ventricles in response to stretching from volume overload. It is used in the assessment of progression of underlying PH and response to treatment. Natriuretic peptides cannot make the distinction between pre- and post-capillary PH as they are elevated in both conditions.

### Associated clinical presentation in NYHA class I-IV

Congestive heart failure (CHF) is categorized from Class I-IV and Stage A-D by NYHA (Table 4). Patients endorse orthopnea, paroxysmal nocturnal dyspnea, palpitations, anginal chest pain, pre-syncope and/or syncope. Physical exam findings include elevated jugular venous pressure, right ventricular heave, loud pulmonary component of S2 and a pansystolic murmur of tricuspid regurgitation. Immediate organ failure includes congestive hepatopathy in which patients report right upper quadrant pain, hepatomegaly, and ascites. Weight gain from peripheral edema due to RV failure and extracellular volume expansion is a frequent presenting feature of PH. Patients' symptoms of dyspnea, lethargy and fatigue are due to inadequate CO during exertion, representing advanced PH with overt RV failure (31). The 6-min walking test (6 MWT) is widely used in assessing decreased exercise tolerance in patients with suspected PH (32). This inexpensive and easily applicable test is a valid measure of symptomatic improvement and has prognostic importance. The correlation with variables of maximal cardiopulmonary exercise test and disease severity markers allows this repeatable standardized test to be used along with other invasive and non-invasive disease severity markers allows this repeatable standardized test to be used along with other invasive and non-invasive disease markers in assessing disease progression and response to treatment (9). ESC guidelines in 2022 proposed a risk-assessment tool including at follow up based on WHO functional class (Table 5), 6 MWT, and NT-proBNP (11).

**Table 4:** Comparison of American College of Cardiology (ACC)/American Heart Association (AHA) Stages and New York Heart Association (NYHA) functional classifications based on patient symptoms and objective assessment (29, 30).

**ACC/AHA stage**	**NYHA functional classification**
Stage A: At high risk for HF but without structural heart disease or symptoms of HF	None
Stage B: Structural heart disease but without signs or symptoms of HF	Class I: No limitation of physical activity. Ordinary physical activity does not cause symptoms of HF
Stage C: Structural heart disease with prior or current symptoms of HF	Class I: No limitation of physical activity. Ordinary physical activity does not cause symptoms of HF
	Class II: Slight limitation of physical activity. Comfortable at rest, but ordinary physical activity results in symptoms of HF
	Class III: Marked limitation of physical activity. Comfortable at rest, but less than ordinary activity causes symptoms of HF
	Class IV: Unable to carry on any physical activity without symptoms of HF, or symptoms of HF at rest
Stage D: Refractory HF requiring specialized interventions	Class IV: Unable to carry on any physical activity without symptoms of HF, or symptoms of HF at rest

HF, heart failure.

**Table 5:** World Health Organization (WHO) classification of functional status of patients with pulmonary hypertension (11, 33).

**Class**	**Description^a^**
WHO-FC I	Patients with PH but without resulting limitation of physical activity. Ordinary physical activity does not cause undue dyspnea or fatigue, chest pain, or near syncope
WHO-FC II	Patients with PH resulting in slight limitation of physical activity. They are comfortable at rest. Ordinary physical activity causes undue dyspnea or fatigue, chest pain, or near syncope
WHO-FC III	Patients with PH resulting in marked limitation of physical activity. They are comfortable at rest. Less than ordinary activity causes undue dyspnea or fatigue, chest pain, or near syncope
WHO-FC IV	Patients with PH with an inability to carry out any physical activity without symptoms. These patients manifest signs of right HF. Dyspnea and/or fatigue may even be present at rest. Discomfort is increased by any physical activity

PH, pulmonary hypertension; WHO-FC, World Health Organization functional class.

a Functional classification of PH modified after the New York Heart Association functional classification according to the World Health Organization (34).

### Poor fluid tolerance in septic patients with pulmonary hypertension

**Figure 2:**
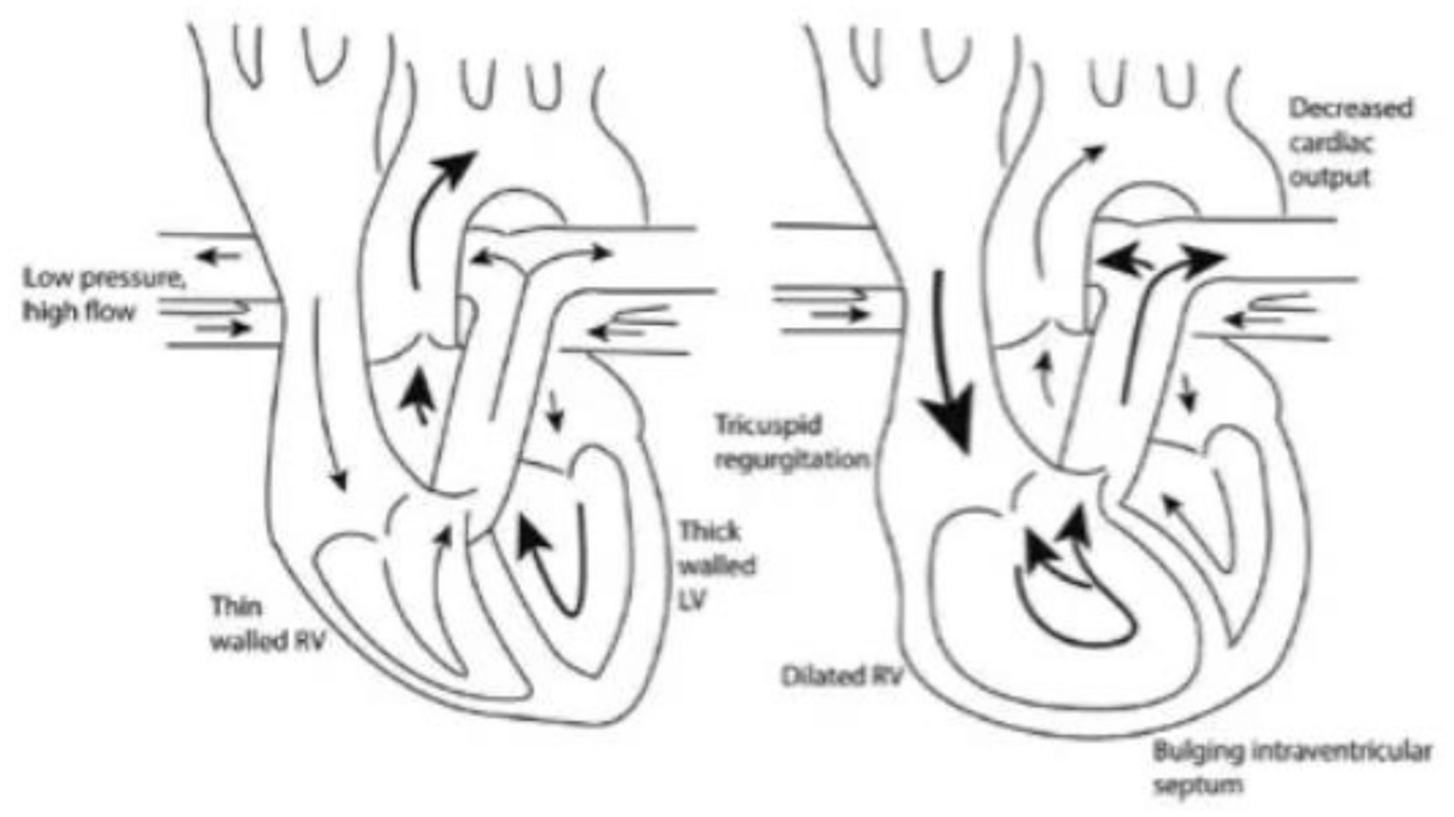
As the right ventricle dilates and causes bowing into the left ventricle, left ventricular filling is decreased resulting in reduced cardiac output (38).

Fluid therapy is a vital part of management of the critically ill patient with sepsis. The rationale behind fluid resuscitation in sepsis is to improve CO and organ perfusion, thereby limiting organ dysfunction. The goal of fluid resuscitation is to increase preload until optimal forward SV is achieved (35). Despite being considered a relatively benign therapy in sepsis, emerging evidence in septic patients with PH has identified that there is a need for conservative fluid balance to avoid excessive morbidity and mortality (36). Currently, there is no existing guidelines on how to resuscitate septic patients with PH. These patients can be extremely challenging to manage in the critical care setting due to the need to volume load in sepsis may worsen shock in a tenuous RV by exacerbating pressure- and volume- overload. Excess volume loading of the RV can lead to RV failure by further reducing RV contractility and left bowing of the interventricular septum can lead to underfilling of the LV and further reduction in CO (Figure 2) (37, 38). Expertise management focuses on treating underlying acute illness, supportive measures with inotropes and vasopressors, judicious fluid management and PH drugs (11).

### Septic cardiomyopathy

Sepsis-induced cardiomyopathy contributes to organ failure and shock. It is multi-factorial, poorly understood, and has both short- and long-term consequences (39). Despite the lack of diagnostic criteria for sepsis-induced cardiomyopathy, it is known to have three characteristics: LV dilatation, depressed ejection fraction, and reversible providing the patient's recovery (40).

Parker et al. described the reversible myocardial dysfunction as the result of myocardium being both functionally and structurally injured by inflammatory cytokines (41). The role of cytokines has been advocated in the genesis of septic cardiomyopathy due to *in vitro* studies having shown that myocardial cell shortening is reduced by exposure to the serum of septic patients (42). Additional factors contributing to the cardiac depression include increased phosphorylation of troponin I reduced myofilament response to calcium (43).

The depressed LV systolic function is not associated with high filling pressures, unlike classic cardiomyopathy, due to RV dysfunction and improvement in LV compliance (44). RV dysfunction is related to PH as the depressed intrinsic contractility due to circulating cytokines act to avoid significant elevation of LV pressure and protects the pulmonary circulation (45).

Goal in treatment of septic cardiomyopathy is to treat the underlying sepsis and provide inotropic support for cardiogenic shock when present.

### Vascular dysfunction in septic patients with pulmonary hypertension

Lastly, in septic patients with PH sepsis is associated with increased endothelial cell expression and activation of the coagulation cascade, which play a vital role in the development of organ dysfunction (46). Endothelial dysfunction induced by sepsis includes shedding of the endothelial-glycocalyx, loss of anticoagulant function, and decreased expression of thrombomodulin (47). In addition, monocytes and leukocyte contribute to activation of the coagulation cascade. The combination of diffuse endothelial injury, paracellular leak, and increased microthrombosis results in capillary hyperpermeability (48). Sepsis-induced vascular dysfunction and lack of response to vasopressor therapy is a vasoplegic state mediated by impairment of vasoconstrictive receptors (49). The increased arterial and venous dilatation due to failure of the vascular smooth muscle to constrict is attributed to increased mediators, including prostacyclin and nitric oxide (NO) produced by endothelial cells. The cellular injury accompanied by proinflammatory mediators progress to organ dysfunction with variable clinical manifestations. The increased production of both natriuretic peptides and NO occurs in arterial dilation resulting in systemic hypotension. The profound vasodilation occurs in cutaneous and splanchnic vascular beds, decreasing venous return and CO (50). Mechanisms underlying organ failure in sepsis have been partially elucidated, with impaired tissue oxygenation playing a dominant role (51). The signs of organ dysfunction may be subtle, thus knowing the warning signs of incipient sepsis can shorten the interval before initiation of treatment (52).

### First line therapy-intravenous fluid

Intravenous fluid therapy remains a highly debated topic in septic patients with PH. The effective fluid management to treat hypovolemia and prevent hypervolemia in these patients remains unclear due to lack of predefined therapeutic goals. Other challenges include heterogeneous nature of PH, variable methods of hemodynamic monitoring, and complex disease processes that present with conflicting goals of management. Accurate assessment of intraventricular volume is imperative to prevent adverse outcomes. Data are lacking regarding the most accurate volume resuscitation endpoints for PH patients, and there is a wide variability of practice between individual physicians and institutions for fluid management (53).

### Predictors of fluid responsiveness

The end-point of fluid therapy is to optimize tissue perfusion, but considering this parameter cannot be directly measured, surrogate end points are used as clinical indicators. These include static and dynamic hemodynamic parameters, serum lactate concentration, capillary refill time, and urine output (10). The end goal with fluid resuscitation is to increase SV by at least 10% in fluid responders (36). Based on the Frank-Starling principle, optimization of preload will result in a constant increase in SV. Fluid administration will increase SV if the increase in mean circulatory filling pressure (MCFP) is greater than the CVP and both ventricles are functioning on the “ascending limb” of the Frank-Starling curve (Figure 1).

Numerous cases have reported that half of hemodynamically unstable patients in the intensive care unit (ICU) are fluid responders (54). The rest of the patients are at risk for the adverse effects of fluid loading when a patient is on the plateau surface of the Frank-Starling curve. The non-linear left ventricular pressure-volume curve is a result of diastolic non-compliance at higher filling pressures (55). As a patient approaches the plateau portion of the curve, atrial pressure increases which leads to increased venous and pulmonary hydrostatic pressures (56). The combined increase in pressures, along with increased natriuretic peptides, result in a shift of fluid from the intravascular to the interstitial space and ultimately leads to pulmonary and peripheral edema (57). Tissue edema impairs oxygen and metabolite diffusion, and distorts capillary blood flow with a profound effect on the venous pressure and function in vital organs (58). In a recent study, Gavelli et al. (59) reported elevated levels of extra vascular lung water (EVLW) being associated with mortality in critically ill patients, thus supporting the detrimental effects of over-resuscitation. The Marik-Phillips curve in Figure 1 further demonstrates the complications of fluid administration in the form of EVLW at the plateau portion of the Frank-Starling curve.

Intravenous fluid (IVF) administration has long been termed the ‘cornerstone of resuscitation' (60). EGDT targeting a specific CVP (>8 mmHg) to guide fluid administration was initially thought to improve survival outcomes in patients with severe sepsis but studies found that only about 50% of these patients were fluid responders (61). The ability of crystalloids to increase the pressure gradient for venous return by expanding intravascular volume was evaluated by Chowdhury et al. It was reported in fluid responders that <5% of fluid bolus remained in the intravascular space by the end of the infusion (62). Although the mean arterial pressure (MAP) initially increased after the fluid bolus, it had returned to baseline in an hour with no increase in urine output. The ARDSnet Fluid and Catheter Treatment (FACTT) trial further assessed the hemodynamic profile an hour after the fluid bolus and concluded only 23% of septic patients were fluid responders with no change in urine output within 1 to 4 h after the fluid bolus (63).

The effects of a fluid bolus in septic patients were measured by Monge-Garcia. His study demonstrated an overall increase in MAP in 44% of patients, 67% of whom were fluid responders (64). A decrease in SVR was also noted which suggests the majority of patients with sepsis are not fluid responders and the hemodynamic changes in the fluid responders are likely to be short-lived and clinically insignificant (65). The increased cardiac filling pressures, damaged endothelial glycocalyx and arterial vasodilation are adverse consequences of fluid resuscitation that can likely increase the morbidity and mortality of patients with sepsis. Restriction of IVF in ICU patients with septic shock did not result in fewer deaths at 90 days than standard IVF therapy (66). The CLOVERS trial showed similar outcomes on 90-day in-hospital mortality in septic patients treated with liberal and restrictive fluid strategy (67).

### End points of resuscitation

The ProCESS, ARISE and PROMISE studies confirmed that there was no survival benefit of EGDT compared to usual resuscitation (68). The mandate of administering 30 ml/kg of IVF for intravenous fluids for hypotension or lactate >4 mmol/L within 3 h of hospital presentation needs to be reconsidered. The majority of septic patients with hypotension do not respond to fluids and this approach likely leads to an increase in the mortality of these patients (69). Furthermore, elevated blood lactate is not likely to be associated with inadequate oxygen delivery and attempts to increase oxygen delivery do not result in increased oxygen consumption or lower lactate concentrations; they do however increase mortality in septic patients in the ICU (70).

The clinical relevance of other possible predictors to fluid responsiveness have been considered. Comparison of static indices of preload include CVP, PCWP, pulmonary artery diastolic pressure (PADP), and inferior vena cava (IVC) diameter. CVP is most commonly utilized because it's easily obtainable in the ICU. CVP is used as a preload index of both ventricles (right more than left). The end-diastole CVP equalized to the end-diastolic pressure of both ventricles, pulmonary artery, pulmonary capillaries, and the left atrium. LVEDP is related to LVEDV thus determining the LV preload. Based on the Frank-Staling law, by increasing the CVP, a higher CO is expected up to a certain threshold. The criticism with using CVP as an endpoint is that in patients with PH, CVP differs significantly from the LVEDP (71). Furthermore, estimates of cardiac preload should enable prediction of fluid responsiveness, which under clinical conditions CVP is unable to meet the demands.

While CVP is considered a static measurement of hemodynamic profile, the idea of dynamic monitoring using stroke volume variation (SVV) and pulse pressure variation (PPV) has gained wide acceptance and popularity. Many studies performed in septic patients in the ICU setting have shown both of these measures to be superior to the more commonly measured static preload variables and CVP (72, 73). Ganter et al. (74) showed the clinical utility and accuracy of dynamic indices as bedside indicators of preload reserve and fluid responsiveness. When analyzing increased CO in a postoperative setting, PPV has repeatedly been shown to be superior to SVV as a reliable predictor of fluid responsiveness; both indices are significantly higher in responders than in non-responders and far superior to CVP and PAP (75). Preliminary results have shown EGDT based on PPV monitoring during high-risk surgery improves postoperative outcome and decreased the length of hospital stay (54). Although algorithms and devices for continuous calculation of PPV and SVV exist, measurements obtained by one specific device may not be applied to indices from other manufacturers (76). Comparison of different automated indices can be reliably achieved if simultaneous measurements are performed in the same patients (77). To date, no such comparison of automated indices is available in the literature.

Vena cava assessment by measures of IVC diameter and collapse has also been proposed as tools for estimating intravascular volume status. Due to the lack of valve between the vena cava and right atrium, fullness of the IVC correlates with increased RAP. Measurement of the IVC diameter during spontaneous respiration correlates with CVP. A change in diameter with spontaneous respiration of >12% has been associated with an increase in CO of >15% after a fluid bolus (78). The pitfall of using IVC diameter as a marker of fluid responsiveness is that readings can be falsely elevated in RV failure and pulmonary embolism. Another general disadvantage of this technique is that findings can be confounded, especially in patients with PH.

In addition to IVC assessment, point of care ultrasound is also used to assess another stroke volume surrogate: left ventricular outflow tract velocity time integral (LVOT VTI). LVOT VTI is a measure of cardiac systolic function and CO. Doppler derived CO is obtained by measuring flow across the LVOT which is determined by the VTI of the Doppler signal directed across the LVOT, multiplied by the cross sectional area of the LVOT and heart rate (79). Given the close correlation between LVOT VTI and Doppler derived CO, LVOT VTI is used as a reliable surrogate for CO in the absence of LVOT abnormalities (80). Diminished LVOT VTI (<10 cm) strongly predicts adverse clinical outcomes and identifies patients who may benefit most from advanced therapies. A study by Ristow et al. evaluated over 900 patients with coronary artery disease and demonstrated increased rates of heart failure hospitalization for subjects within the lowest VTI quartile (81). LVOT VTI provides enhanced prognostic information over ejection fraction, as it focuses on forward CO (80). Low CO is a known precursor to cardiogenic shock and multi-organ dysfunction. The accuracy of Doppler derived CO is limited by errors in determining the cross sectional area of the LVOT; utilizing LVOT VTI rather than Doppler derived CO eliminates this source of error (82).

Monitoring of RA and SVC central venous oxygen saturation was thought to be an indicator of the quality of care delivered to septic patients, when the value is above 65%. Considering the oxygen saturation of most septic patients in the ICU is either within normal limits or increased, this recommendation is no longer endorsed as a method to guide fluid resuscitation (83). The Process, Arise and Promise trials further demonstrated that monitoring of venous oxygen saturation in patients with sepsis has no scientific basis or overall benefits (84).

The Surviving Sepsis Campaign guideline by Dellinger recommended using lactate as end point of resuscitation (59). It was thought that an elevated lactate was the result of tissue hypoxia; when compared to usual care however, Hotchkiss and Karl debunked this notion by demonstrating that the sympathetic response is sepsis causes a catecholamine surge which stimulate the Na/K ATPase activity resulting in elevated levels of lactate (85). The ANDROMEDA-Shock clinical trial dichotomized patients in septic shock based on their fluid responsive status and sought to compare target serum lactate levels with a resuscitation strategy targeting normalization of capillary refill time. The analysis found no significant difference in outcomes between the two groups, including a reduction in all-cause 28-day mortality (84).

PCWP is a reasonable surrogate marker for LVEDP as it's an integrated measurement of the compliance of the left side of the heart and the pulmonary circulation; it allows clinicians to titrate vasoactive medications and pulmonary vasodilators. PCWP is used to evaluate volume status to guide fluid administration during septic shock, where the PCWP goal is between 12 and 14 mmHg (86). An alternative guide for treatment is PAP, which are amenable to continuous monitoring. In patients with normal LV function, PADP is an accurate reflection of LVEDP, but the validity of using PADP as a measure of LV filling pressure in patients with septic shock is still a matter of some debate (87). Bouchard et al. found increases in systemic pressure produced a consistently larger increase in LVEDP than PADP. Atrial pacing in these studies resulted in a consistent disparity in pressures; large LV “a” waves were not observed in patients with elevated pulmonary arterial pressures (88).

Adequate urine output of >0.5 mL/kg/hour in adults has been promoted as a possible resuscitation strategy to normalize tissue perfusion. This endpoint, however, has been brought into question due to the lack of correlation between urine output and physiologic variables (89). Urine output was unable to identify fluid responders after a fluid challenge (90). Diuresis is a poor endpoint that may lead to over or underestimation of fluid resuscitation. As urine output is recognized as a poor resuscitation target, other resuscitation protocols including targets and endpoints that can be obtained with non-invasive hemodynamic monitoring devices are needed in clinical practice to guide resuscitation strategies.

In summary, in septic patients with PH, serial lactate measurement, capillary refill time, and urine output, though with their own limitations, are useful adjunctive end point in resuscitation to pressure-guided modality. When pressure monitor is utilized, continuous, dynamic variations are more useful than static measures in guiding fluid therapy. Individualized plans for each PH patient should be assessed for hemodynamic optimization of sepsis. Pulmonary artery catheter can be of great utility to guide fluid management as it records CVP and PCWP continuously. Point of care echocardiography measuring LVOT VTI represents a newer non-invasive method in CO assessment that requires skills in ultrasonographic performance and interpretation.

## Challenges and recommendations in IVF resuscitation in pulmonary hypertensive patients with sepsis

### Heart failure

Patients with PH are at risk for right heart failure further complicated by volume overload. Precipitating causes for volume overload include PH severity and degree of end-organ dysfunction associated with acute illness. Fluid management in heart failure patients is often difficult, as patients with predominantly diastolic dysfunction of the RV and elevated filling pressures can have systemic hypotension with subsequent release of antidiuretic hormone (91). Short-term diuretics have been considered but patients are likely to have worsening renal function, especially with underlying critical illness. Initiation of pulmonary vasodilators is often required in acute decompensated RV failure so as to avoid systemic hypotension and worsening tissue perfusion (92).

### Other end organ failure

Progressively elevated PVR leads to elevated RAP which can precipitate hepatic congestion resulting in ascites (93). Renal congestion combined with poor renal perfusion can also occur, which leads to diuretic resistance and worsening renal function due to prerenal azotemia. Cardiorenal syndrome is the result of reduced CO contributing to reduced arterial renal perfusion. The complexity of treatments to improve volume status and organ perfusion often times requires intravenous diuretics. In addition, severe cases of sepsis may require dopamine or dobutamine to improve CO and renal perfusion before effective diuresis is achieved.

### Hypoperfusion of tissue from interaction of pathophysiology between sepsis and pulmonary hypertension

Sepsis poses a myriad of physiologic derangements including increased vasodilation, hypovolemia and decreased SVR that must be overcome by an increment in CO (94). Low SVR leads to decreased RV coronary perfusion and an increase in PVR resulting in decreased RV output. Due to the increased PVR, increasing CO may prove very difficult in patients with PH, and sepsis can trigger acute RV failure (95). Considering pulmonary arterial hypertension imposes an increased afterload to the RV and RV dysfunction being a major determinant of the outcome of sepsis, PH leads to a more severe manifestation of shock than non-PH septic patients (96). The limited ability of the RV to increase CO to compensate for the decrease in SVR in septic shock makes it difficult to deliver oxygen to the organ and tissues. Intravascular volume replacement and vasoactive drugs have limited ability to meet increased metabolic demands. Fluid resuscitation should not be liberal in septic patients with PH and sepsis and should be guided by invasive hemodynamic monitoring devices. The use of pulmonary vasodilators in sepsis is reserved to cases where further decrease in PVR is needed to improve CO and systemic perfusion. However, caution must be exercised to prevent hemodynamic collapse as these agents produce systemic effects that could potentially worsen the patient's overall condition (10).

### Alternative treatment with diuretics

Chronic fluid retention due to PH related right ventricular failure is typically treated with loop diuretics. Diuretics diminish pleural effusions, hepatic congestion, peripheral edema, and decrease the interventricular septal deviation thus improving left ventricular output. Preload reduction in PH has been shown to improve LV filling and CO by optimizing diastolic ventricular interdependence between both ventricles (97). However, excessive diuresis may result in volume depletion and hypotension precipitating an acute cardiogenic shock due to RV failure. Throughout the course of PH, patients may require adjustments to diuretic therapy based on disease progression and severity. Increasing doses of diuretics may be needed with worsening right heart failure and cardiorenal syndrome. Decreasing doses of diuretics may be required with worsening renal function due to volume depletion. Therefore, conservative fluid strategy may need to be exercised in septic patients with PH. The CLOVERS trial evaluated fluid treatment strategies to determine the impact of a restrictive fluids strategy as compared to a liberal fluid strategy. The trial outcomes were similar in both strategies with a lack of significant difference in 90-day mortality rates (68), though there was no subgroup analysis focused on patients with PH. Fluid administration can have deleterious effects by causing edema within vital organs, warranting use of diuretics so as to avoid further organ dysfunction and impairment of oxygen delivery. Conversely, a restrictive fluids approach relies on vasopressors to reverse hypotension and maintain perfusion while limiting the administration of fluid (98). Hence, combined gentle diuresis and restrictive fluid strategy may be considered in septic patients with PH showing signs and symptoms of RV failure.

### Additional therapy with inodilators, nitric oxid, and vasopressin

Levosimendan, nitric oxide, and vasopressin have unique biochemical properties and are clinically efficacious in supportive care of sepsis patients with PH.

Calcium sensitizers, such as levosimendan, are often used to treat PH as the reduction in PAP significantly improves CO and may result in less systemic hypotension. Its mechanism of action is complex and consists of inotropy, vasodilation, and cardioprotection (99). Through reduction of PVR, the pulmonary vascular tone is enhanced by endothelin-1. The levosimendan-induced relaxation results in formation of cyclic guanosine monophosphate (cGMP) and cyclic adenosine monophosphate (cAMP), suggesting an improvement in pulmonary hemodynamics in states of elevated PVR, such as PH. Meta-analysis by Qiu shows that levosimendan improves RV function during 24-h infusion in treating RV failure in patients with a variety of heart and lung disease (100). Although consistent evidence of prophylactic levosimendan improving all-cause mortality is lacking, levosimendan is approved for acute heart failure as PH often represents a frequent co-morbidity (101). Its performance in cardiogenic shock was found to be superior to dobutamine in improving RV function and reducing PAP (102, 103). When used in sepsis and septic shock, meta-analysis by Chang fails to show any mortality benefits though reports significantly elevated cardiac performance and lactic acid normalization (104). Large scale randomized controlled trials are needed to provide evidence for levosimendan use in septic patients with PH.

In cases of pre-capillary PH, inhaled NO is the agent of choice for diagnosing vasoreactivity during right heart catheterization. Its long term use in adults has remained an investigational and no data yet to support its efficacy (11). However, approved therapy for pulmonary arterial hypertension (PAH) consists of drugs (sildenafil, tadalafil, or riociguat) that enhance the nitric oxide-cGMP biological pathway (105). Local delivery of this potent pulmonary vasodilator will result in decreased perfusion of poorly ventilated lung and intrapulmonary shunting. The overall improvement in ventilation-perfusion matching with minimal systemic effects is in stark contrast to other systemically administered agents (106). The effects of sepsis on endothelial dysfunction can be quantified by endothelial NO bioavailability; the excessive NO and absolute deficiency of vasoactive hormones result in refractory vasorelaxation (107). Although gas exchange improves with inhaled NO with minimal systemic sequelae, the effect is not consistent and the potential impact on morbidity and mortality in septic patients with PH is not well-studied.

Vasopressor therapy is often required in combination with fluids to augment MAP. Vasopressin, which is stored and released from the posterior pituitary, is relatively deficient in sepsis. It acts on V1 and V2 receptors, causing vascular smooth muscle contraction and water reabsorption in the collecting tubules of the kidney (108). It augments SVR without increasing PAP or PVR. In selective vascular beds, including coronary, pulmonary and cerebral vasculature, low dose vasopressin is found to have vasodilatory effects (109, 110). In patients with sepsis, combination of vasopressor therapy (e.g., catecholamine + arginine vasopressin) yields to better outcome for refractory shock (111). Given the lack of robust evidence demonstrating improved outcomes with vasopressin, it is often not considered as initial management involving treatment of septic patients with PH. It could be considered as an adjunct to existing catecholamine therapy in refractory shock while monitoring its efficacy through continuous MAP recording on an arterial line.

### Clinical outcome

**Figure 3:**
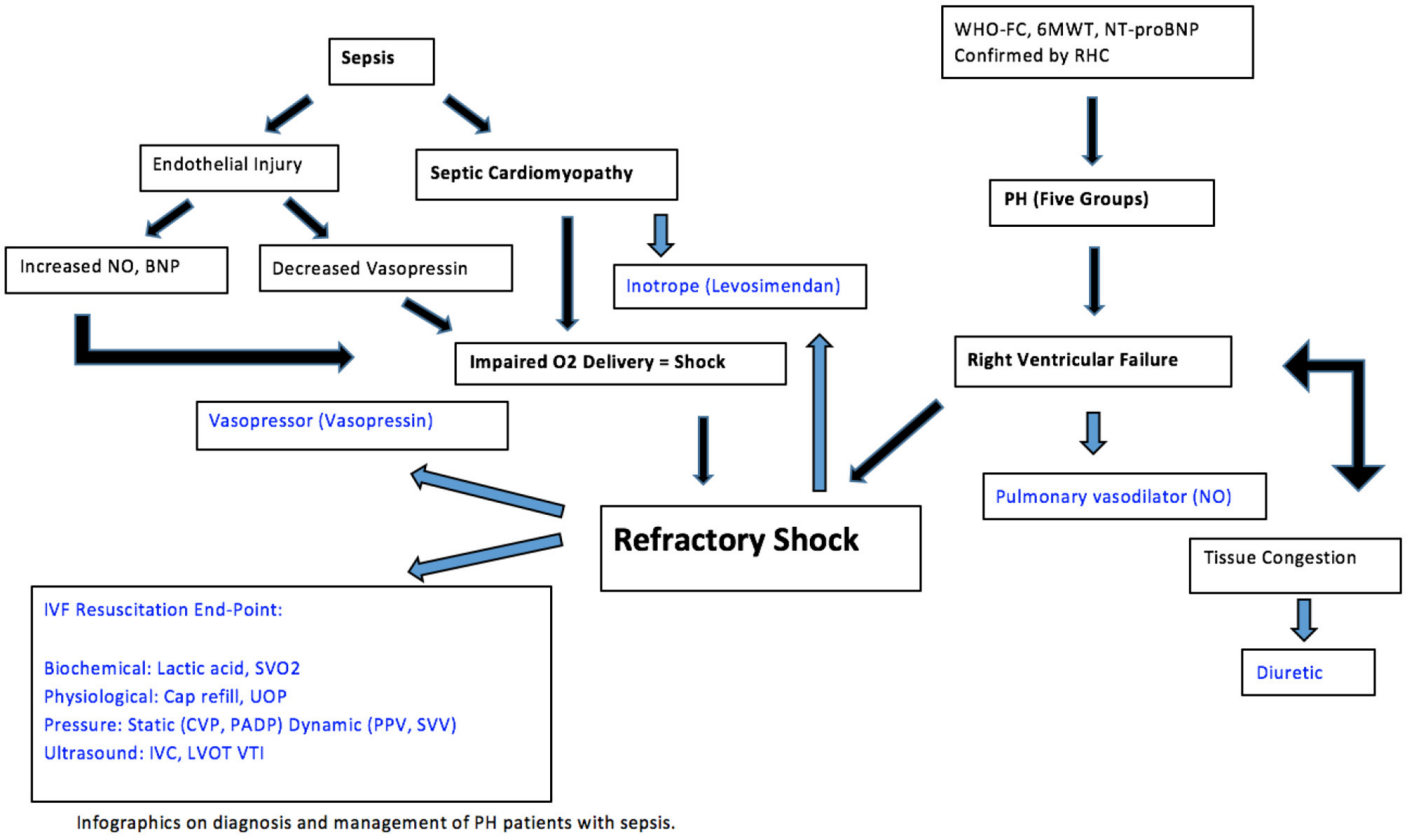
Various pathological pictures and the appropriate therapeutic suggestions.

Despite advances in medical therapy, pulmonary hypertension and sepsis remain lethal conditions for many patients. National surveillance data reported mortality rated from PH have increased from 5.2 to 5.4 per 100,000 over a 22-year period (112). With PH progression, the marginalized RV is susceptible to failure due to fluid depletion or excess. The principles of care are focused on improving RV function and tissue perfusion to ensure oxygen delivery to end organs, with or without sepsis. Given the most up to date evidence, therapeutic options include conservative fluid strategy, inotrope, pulmonary vasodilators, and gentle diuresis, guided by end points of resuscitation (Figure 3). When sepsis is present, it is important to treat the underlying cause while applying the above-mentioned general principles in caring for patients with PH.

## Author contributions

All authors listed have made a substantial, direct, and intellectual contribution to the work and approved it for publication.
